# Idiopathic Intracranial Hypertension – How Can Orthoptists Help Improve Care?

**DOI:** 10.22599/bioj.427

**Published:** 2024-12-06

**Authors:** Daisy MacKeith

**Affiliations:** 1 Cambridge University Hospitals NHS Foundation Trust, UK

**Keywords:** Idiopathic intracranial hypertension, Weight stigma, Obesity, Fulminant IIH, Internalised weight stigma, Anti-fat bias, Functional vision loss, Non-organic vision loss

## Abstract

Care for people with idiopathic intracranial hypertension (IIH) needs improving and I think orthoptists are in a perfect position to help.

**The problems with IIH care are multi-fold:**

- People with fulminant IIH are still losing sight due to delayed diagnosis or mismanagement.
- People with IIH often have a poor quality of life due to disabling chronic headaches and poor mental health.
- There is a lack of access to evidence-based weight-loss interventions and support.
- Weight stigma in healthcare remains pervasive and damaging and disproportionately impacts women.
- Functional vision loss and headaches in IIH can complicate the interpretation of visual function which can have implications for management.
- There is a lack of evidence to support treatment options.

Below I will describe the issues in more detail and outline the ways in which we as orthoptists can help this patient group.

Dear Editor,

Care for people with idiopathic intracranial hypertension (IIH) needs improving and I think orthoptists are in a perfect position to help.

**The problems with IIH care are multi-fold:**

- People with fulminant IIH are still losing sight due to delayed diagnosis or mismanagement.
- People with IIH often have a poor quality of life due to disabling chronic headaches and poor mental health.
- There is a lack of access to evidence-based weight-loss interventions and support.
- Weight stigma in healthcare remains pervasive and damaging and disproportionately impacts women.
- Functional vision loss and headaches in IIH can complicate the interpretation of visual function which can have implications for management.
- There is a lack of evidence to support treatment options.

Below I will describe the issues in more detail and outline the ways in which we as orthoptists can help this patient group.

## Fulminant IIH

Fulminant IIH is a subset of IIH and is defined as IIH presenting with severe papilloedema and rapidly declining visual function within one month of diagnosis. Fulminant IIH requires aggressive management and must be recognised quickly (Thambisetty *et al*., 2007).

Permanent sight loss can be prevented by lowering intracranial pressure through neurosurgical interventions such as shunt insertion or venous sinus stenting. Optic nerve sheath fenestration can also be used to prevent permanent sight loss (Scherman *et al*., 2018). There is a lack of evidence for which is the safest and most effective surgical intervention, but a randomised controlled trial to compare shunts and stents is due to be completed in 2027 (see Sinclair *et al*., 2023 protocol).

Permanent sight loss can occur when there is a delay to diagnosis or a delay to intracranial pressure (ICP) lowering intervention (Mollan *et al*., 2018) and in my experience this is still happening in practice. However, there seems to be a lack of published evidence to reflect this, and we therefore have work to do in terms of auditing current services against the consensus guidelines and publishing cases of permanent sight loss in IIH.

Care at presentation often involves primary care (general practice), high street optometrists, emergency department staff, ophthalmologists, neurologists and neurosurgical teams. Orthoptists are more likely to meet these patients further along in their journey but that must not stop us advocating for an improvement to care at presentation and the prevention of avoidable sight loss. Auditing your own hospital against the consensus guidelines is a great place to start.

## Headache burden, mental health and quality of life

People with IIH often have persistent chronic headaches following resolution of papilloedema and normalisation of ICP (Mollan *et al*., 2021) which has a significant impact on quality of life. There is an urgent need for improved evidence-based headache treatments specific to IIH (Mollan *et al*., 2021). Patients often feel that their headache symptoms are not addressed. As orthoptists we can help by ensuring there are robust patient pathways for IIH which include neurology, for patients who require headache management.

There is a high association between having a rare disease and poor mental health and IIH is no exception (Kleinschmidt *et al*., 2000; Spencer-Tansley *et al*., 2022). Spencer-Tansley *et al*. (2022) systematically explored the mental health burden experienced by those with a rare disease and their family members and concluded that the cause of mental health issues was often specific to managing a condition that is rare; their published recommendations included equipping healthcare professionals with the skills and knowledge to demonstrate awareness of the burden of living with a rare disease, undertake sensitive conversations about mental health and sign-post people to support. The authors also recommend improving access to mental health services. As per Naylor *et al*., 2012, there is evidence that integrating mental health support with chronic disease management is necessary to improve outcomes for people who live with both physical and psychological conditions. At present, access to NHS psychological services is limited due to service pressures (Bell and Pollard, 2022) but orthoptists can encourage those who are struggling to access what is available via their general practitioner (G.P.) or employer. Orthoptists can lobby for better access to mental services and better integrated services for people with IIH. Furthermore, orthoptists can also signpost people with IIH to the IIHUK website for patient information leaflets and to the Migraine Trust website for life-style advice to improve headache symptoms (IIHUK, 2024; The Migraine Trust, 2021).

## IIH – an issue of equality, diversity and inclusion

IIH predominantly affects women with a high or very high BMI and therefore patients face oppression due to these two intersecting marginalised identities. The issue of weight-stigma and sexism cannot be ignored in IIH. If we ignore it, we may inadvertently discriminate and perpetuate damaging attitudes. Obesity is a complex issue involving genetics and epigenetics, environment, lifestyle, psycho-social factors, inequality and more (Bray *et al*., 2017). Anti-fat attitudes remain pervasive, and many people view obesity as a moral failing; this moralisation of obesity is a complex issue which needs exploring in order to change attitudes and improve outcomes (Ringel and Ditto, 2019).

In addition to the externalised oppression experienced by patients with IIH there is often internalised oppression; negative societal attitudes and stereotypes can be absorbed resulting in low self-esteem, body dissatisfaction, lower physical activity, disordered eating and avoidance of healthcare (Puhl and King, 2013). We know from studies of other health conditions associated with obesity that patients can internalise a sense of failure and feel blamed for their weight and health condition by health professionals (Salvia *et al*., 2023). Women experience more weight stigma than men and are more likely to internalise weight stigma (Kyle and Pearl, 2024). Furthermore, exercise, healthy eating and self-care can be hard to prioritise due to family caring commitments (Carter-Edwards *et al*., 2009; Andajani-Sutjahjo *et al*., 2004), work and disabling chronic headaches (Mollan *et al*., 2021). Obesity and IIH disproportionately affect people from lower socio-economic backgrounds (Penman and Richey, 2021; Mollan *et al*., 2019) which only adds to the multiple marginalised identities a person with IIH may embody. Childhood trauma such as neglect or abuse may increase the likelihood of obesity and women with a BMI of 40 or more are more likely to report sexual abuse than those with a lower BMI (Wadden *et al*., 2006).

A recent survey of people with IIH found that the majority had a poor experience of discussions about weight with health professionals (Abbott, Denton *et al*., 2023). The gap between the scientific understanding of what causes obesity and public perception (including in a health care setting) perpetuates weight stigma (Abbott, Shuttlewood *et al*., 2023). As orthoptists we can educate ourselves and our colleagues about these issues.

Bariatric surgery has been shown to be more effective than community weight loss programmes in IIH but access to bariatric surgery remains limited (Mollan *et al*., 2021; Abbott, Chan *et al*., 2023)

GLP-1 agonists have a potential role in IIH treatment by lowering ICP (Mitchell *et al*., 2023) and improving symptoms (Krajnc *et al*., 2023) but in practice these medications are in high demand and short supply (Diabetes UK, 2024).

There is a real need for more evidence-based sustainable weight-loss advice for patients with IIH and it would be helpful to seek advice and collaborate with colleagues in dietetics. Unfortunately, this may be difficult in practice due to the service demands faced by dieticians (BDA, 2020). Orthoptists can at least sign-post patients to available community weight-management services or encourage GPs to refer to tier-3 weight management services if appropriate.

## Functional (non-organic) vision loss and diplopia in IIH

A significant minority of patients with idiopathic intracranial hypertension have co-existing functional (non-organic) vision loss (Ney *et al*., 2009). Interpretation of visual fields can be complicated by both FVL and because visual field defects can occur in migraine and headaches, particularly during an attack (Yener and Korucu, 2017). Furthermore, migraine can cause cognitive symptoms which may affect the reliability of subjective vision test results (Vuralli *et al*., 2018). Orthoptists are trained and experienced both in detecting FVL and in assessing and interpreting visual fields so are well placed to help the wider team by providing an interpretation of both subjective and objective test results. Where there is a discrepancy between the degree of papilloedema and the degree of subjective vision test results, co-existing FVL may be suspected and investigated.

There has been recent progress in the understanding of how best to approach FVL; Ramsay *et al*. (2024) advocate a frank discussion with the patient in which the FVL is labelled and explained with an emphasis on the evidence that the patient’s potential vision is demonstrably better than they are experiencing. Where FVL has been demonstrated, it is important that any treatment to lower ICP is guided by the presence and severity of papilloedema or by lumbar puncture/lumbar infusion study results.

Diplopia was reported by 18% of participants in the IIH Treatment Trial, but only 3% were found to have a sixth nerve palsy on examination; thus it may be due to the existence of transient sixth nerve palsy occurring during spikes in ICP (Wall *et al*., 2014). Other cranial nerve palsies have also been reported but are extremely rare (Chen *et al*., 2021) and all patients should undergo urgent neuroimaging at presentation to rule out other causes (Mollan *et al*., 2018). Additionally, convergence insufficiency may be associated with, or exacerbated by, migraine and research in this area may be warranted (Singman *et al*., 2014). Orthoptists have an important role in assessing eye movements, managing diplopia and ensuring that the presence or absence of cranial nerve palsies is documented and communicated to the wider team.

## National consensus guidelines for IIH

In 2018, national consensus guidelines for IIH were published (Mollan *et al*., 2018) in a bid to standardise and improve care. Whilst there remains a lack of high-quality evidence for treatment in IIH, these consensus guidelines drew on multidisciplinary expertise and provide a useful benchmark against which services can be audited. The guidelines include advice on the timing and type of neuroimaging (MRI or CT Head within 24 hours plus MR or CT venography within 24 hours), the use of lumbar puncture, the importance of case history and full blood count to exclude secondary causes such as anaemia. Neurosurgery is also recommended in the event of declining visual function in order to prevent permanent vision loss. The authors advocate for referral to community or hospital-based weight management programme where the BMI is over 30 and for sensitive weight-loss discussions. In terms of headache, the authors advise determining the phenotype and tailoring treatments accordingly, warning of the risk of medication over-use headache and introducing migraine preventative medication early. Additionally, they state that the teratogenic effects of medications used in IIH such as acetazolamide and topiramate must be discussed. They recommend that the burden of living with a rare disease is acknowledged and that patients should be sign-posted to appropriate support and lifestyle advice. The above extracts are by no means exhaustive and all orthoptists working with people with IIH should read the consensus guidelines in full and consider auditing their trust against the recommendations outlined in the paper.

## What can orthoptists do to help?

The below list includes a handful of ideas for improving care for IIH but this is just a starting point. My hope is that more of us will extend our roles in this area and bring fresh eyes to the problems discussed above so that we can collaborate to find solutions.

**Suggested actions:**

- Audit your hospital trust against the consensus guidelines published by Mollan *et al*. (2018).
- Listen to people with IIH compassion and without judgement.
- Recognise and acknowledge the weight stigma experienced by people living with IIH and obesity.
- Report incidents of harm (e.g., sight loss) both locally and regionally.
- Work closely with neurology and neurosurgery to provide timely ophthalmic findings in order to guide management.
- Ensure people with IIH who experience chronic headaches have access to headache management advice from neurology.
- Carry out research on IIH.
- If your trust requires you to have an equality, diversity and inclusion (E.D.I.) objective as part of your appraisal, do something relating to IIH.
- Collaborate with each other to share IIH patient pathways, competency training documents for neuro-orthoptists, etc.
- Lobby for access to psychological support services for people with IIH.
- Lobby for improvements to obesity services for people with IIH.
- Recognise, diagnose and document co-existing functional vision loss and make sure that this is communicated to the patient and wider team.
- Signpost people with IIH to IIHUK and The Migraine Trust websites.

Yours faithfully,

Daisy MacKeith
